# Personalized genomic landscape of prostate cancer in African American men reveals novel drivers and therapeutic opportunities

**DOI:** 10.1371/journal.pone.0360389

**Published:** 2026-10-14

**Authors:** Shanta Murthy, Katie Fago, James Knight, Keith Kowalczyk, Ryan Hankins, Gaurav Bandi, Anatoly Dritschilo, Mira Jung

**Affiliations:** 1 Department of Biochemistry & Molecular Cell Biology, Georgetown University Medical Center, Washington, District of Columbia, United States of America; 2 Department of Genetics, Yale University School of Medicine, New Haven, Connecticut, United States of America; 3 Department of Urology, Georgetown University School of Medicine, Washington, District of Columbia, United States of America; 4 MedStar Georgetown University Hospital, Washington, District of Columbia, United States of America; 5 Department of Radiation Medicine, Georgetown University School of Medicine, Washington, District of Columbia, United States of America; Shaheed Rajaei Cardiovascular Medical and Research Center: Rajaie Cardiovascular Medical and Research Center, IRAN, ISLAMIC REPUBLIC OF

## Abstract

African American (AA) men experience disproportionately high prostate cancer incidence and mortality. Despite this disparity, current genomic repositories are predominantly derived from European populations, and studies using archived tissues are often limited by stromal contamination. We sought to characterize the high-resolution somatic mutational landscape of AA prostate cancer using a high-fidelity, patient-matched model system to identify population-specific drivers and therapeutic targets. We performed whole-genome sequencing (WGS) on 10 matched pairs of primary cell cultures derived from prostate tumors and adjacent non-malignant tissues of AA patients. Our analyses revealed a complex, heterogeneous landscape characterized by significant genomic instability, including frequent chromothripsis and kataegis signatures. Mutation hotspots were concentrated on chromosomes 1, 2, 3, and X, with the highest mutation density on chromosome 17. Functionally impactful mutations were identified in critical oncogenic pathways, including chromatin remodeling (CHD1, ARID1A, ATRX, KDM6A) and DNA damage response (ATM, BRCA1/2, RAD51). Additionally, we identified potentially novel candidate mutations unique to this AA cohort, distinct from those in European-centric datasets. This study provides a foundational, patient-specific WGS dataset derived from African American prostate cancer cell models. The identification of high-frequency actionable alterations in epigenetic and DNA repair pathways highlights the utility of this renewable resource for developing tailored therapeutic interventions and addressing racial disparities in prostate cancer outcomes.

## Introduction

Prostate cancer remains the most frequently diagnosed solid tumor in men, characterized by profound racial and ethnic disparities in outcomes [1]. In the United States, African American (AA) men face a significantly higher incidence of the disease and a mortality rate more than double that of Caucasian American men [2–7]. This disparity persists even in nonmetastatic settings, suggesting that while socioeconomic and environmental factors are contributory, distinct biological drivers likely underpin the aggressive tumor phenotypes observed in this population [3–5,8]. Consequently, identifying these molecular determinants is critical for the equitable application of precision oncology [9,10].

Despite the urgent need for ancestry-informed genomic data, current understanding is heavily skewed. Large-scale repositories, such as The Cancer Genome Atlas (TCGA), are predominantly derived from patients of European ancestry (~90%) [11,12]. Although limited data have pointed to recurrent mutations in genes such as *SPOP, FOXA1, TP53*, and *TMPRSS2* in AA men, broader pan-ethnic analyses often fail to capture population-specific structural variants or DNA repair defects due to the overwhelming representation of European samples [13].

Furthermore, technical limitations have historically hampered genomic discovery in this demographic. Studies using archived formalin-fixed paraffin-embedded (FFPE) tissues often encounter DNA degradation and stromal contamination [14,15], which can confound the detection of true somatic variants. To overcome these barriers, we developed a high-fidelity model system comprising matched pairs of primary epithelial cell cultures derived from fresh radical prostatectomy specimens of AA men. By expanding these cells via conditional reprogramming [16,17], we created an isogenic framework—matching tumor-derived cells with adjacent nonmalignant cells—that serves as a robust internal control for germline subtraction.

In this study, we present the whole-genome sequencing (WGS) analysis of these paired primary cultures. We report a complex mutational landscape characterized by high mutation density on chromosome 17, widespread genomic instability (evidenced by chromothripsis and kataegis), and actionable alterations in chromatin remodeling and DNA damage response pathways. This dataset serves as a foundational resource for identifying novel therapeutic targets and addressing longstanding health disparities in prostate cancer.

## Materials and methods

### Patient cohort, tissue, and data collection

The purpose of this study is to establish cell lines for use in prostate cancer health disparities studies. Patients undergoing elective prostatectomies were enrolled in this study under an Institutional Review Board–approved protocol (IRB Study Number: 2017−0614), underwent preoperative physical examinations, and biopsies to confirm a cancer diagnosis. This was a prospective, IRB-approved clinical trial to obtain annotated clinical samples from African American patients. IRB-approved Informed Consent for Clinical Research was obtained at the time of enrollment. All data were fully anonymized before accessing them.

The fresh specimens were obtained from prostatectomies performed utilizing the DaVinci robotic system. Clinical specimens were immediately transferred from the operating room to pathology. A board-certified pathologist identified and dissected tumor regions based on anatomical mapping and pathological grading (adenocarcinoma) versus non-tumor regions. Non-tumor samples were collected from areas of the prostate not visibly affected by malignancy within the same patient to serve as matched controls. Specimens were stored in media on ice and transported for immediate processing.

### Establishment of paired primary cell cultures

Paired tumor and non-tumor tissues underwent mechanical dissociation and were plated in collagen-coated dishes using keratinocyte serum-free medium supplemented with 5% fetal bovine serum (FBS). Primary cells were cultured for up to four passages. To expand the primary epithelium, we utilized Conditional Reprogramming (CR) technology as previously described [16,17]. Briefly, irradiated 3T3 J-2 mouse embryonic fibroblasts (30 Gy) were used as a feeder layer. J-2 cells were maintained in complete DMEM with 10% heat-inactivated FBS. Primary human epithelial cells were cocultured in a medium comprising complete DMEM and Ham’s F-12 nutrient mix (3:1) supplemented with hydrocortisone, epidermal growth factor, bovine insulin, cholera toxin, amphotericin B, gentamicin, and a ROCK inhibitor (Y-27632). The detailed protocol for establishing cell cultures is available at the following link; https://www.protocols.io/file-manager/9148D4BE307811EDA5460A58A9FEAC02.

### Whole-genome sequencing and alignment

Genomic DNA was isolated from 5 x 10^6^ cells using the DNA Purification Kit (Promega). Whole-genome sequencing (WGS) was performed at the Yale Center for Genome Analysis (West Haven, CT) (RRID:SCR_016637). Following quality control (QC), libraries were sequenced with 151-bp paired-end reads on an Illumina platform. QC parameters were monitored using Illumina’s Sequence Analysis Viewer. Post-run assessment required samples to possess a percentage of bases ≥Q30 and generate at least 90% of the expected number of reads. Samples were demultiplexed using bcl2fastq2 v2.19.0 (Illumina Casava Pipeline) [18]. Passing samples met thresholds of ≥110 Gb total output, ~ 30X mean coverage, and ≥ 95% coverage at 10X.

To filter mouse sequences originating from the feeder layer, paired-end reads were aligned separately to mouse (mm39) and human (hs38DH) reference genomes using BWA-MEM (RRID:SCR_022192) [19]. Reads with higher alignment scores for the mouse reference were strictly removed from the dataset. We independently mapped raw paired-end reads to the mouse (mm39) and human (hs38DH) reference genomes using BWA-MEM. Reads were filtered based on the BWA-MEM-generated AS (Alignment Score) tag: AS_human_ - AS_mouse_ > 10. Any read pair where AS_mouse_ ≥ AS_human_ or where the alignment score difference was ≤ 10 was removed using a custom Python filter script prior to GATK variant calling. This strategy was verified post-calling by performing NCBI BLAST validation on candidate somatic variants to ensure zero residual mouse read contamination.

### Variant calling and annotation

Filtered reads were processed using GATK 4 Best Practices3 (RRID:SCR_001876). Germline variants were identified by aligning to hs38DH, marking duplicates (Picard), and performing joint calling via HaplotypeCaller and GenotypeGVCFs [18–20]. Somatic variants were called using GATK MuTect2, utilizing the matched non-tumor sample as the germline control. Variants were annotated using ANNOVAR (RRID:SCR_012821) [21] and the Ensembl Variant Effect Predictor (VEP) (RRID:SCR_005194) [22], incorporating databases such as ClinVar (RRID:SCR_006169), COSMIC (RRID:SCR_002260), and GO. Somatic mutations were further validated using BLAST (NCBI) (RRID:SCR_011824) and the UCSC Genome Browser (LiftOver) (RRID:SCR_005780) (https://genome.ucsc.edu) to ensure no residual mouse contamination.

### Genomic instability and structural variant analysis

Tumor mutational burden (TMB) was calculated as the number of nonsynonymous somatic mutations per megabase (Mb) of the coding genome [23,24]. Copy number variation (CNV) [25] was analyzed using CNVkit with tumor-normal matching; log2 ratios were segmented to identify significant amplifications and deletions and intersected with the COSMIC Cancer Gene Census.

Chromothripsis was defined by the presence of mutation clusters within single chromosomes, complex rearrangements, or oscillating CNV states. Kataegis was identified by localized hypermutation clusters (C > T or C > G substitutions in cytosine-rich motifs) near rearrangement breakpoints.

### Statistical analysis and visualization

All statistical analyses were executed using R (v4.3.x)/ GraphPad Prism (v10). Normality was evaluated using the Shapiro-Wilk test. Continuous clinical variables were summarized using mean ± standard deviation (SD) and median with interquartile range (IQR), as appropriate. Categorical clinicopathologic variables were summarized as frequencies and percentages. Because tumor-derived and non-tumor samples were obtained from the same patients, paired comparisons were performed using the Wilcoxon signed-rank test**.** Associations between genomic and clinicopathologic variables were evaluated using Spearman rank correlation**,** which was selected because of the small cohort size and the ordinal or non-normally distributed nature of several variables. Genotype–phenotype analyses evaluated relationships between measures of genomic instability and clinical features, including preoperative PSA, Grade Group, and pathologic T stage. Where exact quantitative TMB and CNV burden values were available, these metrics were analyzed similarly. Multiple-comparison correction was performed using the Benjamini–Hochberg false-discovery-rate method where appropriate. All statistical tests were two-sided. Exact P values are reported, and P < 0.05 was considered statistically significant for prespecified analyses. Given the exploratory nature and limited sample size of the cohort (N = 10), effect sizes, correlation coefficients, and directionality were emphasized, and analyses were interpreted as hypothesis-generating rather than confirmatory. Differences in mutational burden between tumor and non-tumor samples were assessed using the Wilcoxon rank-sum test. Pathway enrichment was evaluated with adjusted P values (false discovery rate < .05). Data visualization was performed using R (v4.2.0) and Python (v3.10) (RRID:SCR_024202). Mutational landscapes were visualized using Mutation Mapper and D3Oncoprint to integrate OncoKB annotations.

## Results

To identify tumor-specific drivers with high specificity, we performed whole-genome sequencing (WGS) on 10 matched pairs of primary cell cultures derived from AA patients. Pathological characteristics included a Gleason grade of 7 (4 + 3 or 3 + 4), PSA levels ranging from 3.4 ng/mL to 13 ng/mL, and tumor stages classified as T2-T3 after prostatectomy (Table 1). This paired design enabled precise subtraction of germline variation and sequencing artifacts by using the non-tumor cells as a patient-specific reference. We focused our analysis on nonsynonymous mutations in coding regions to identify functionally significant drivers and validated our findings against major genomic repositories. The overall cohort summary shows the following means and IQRs. The median BMI (kg/m^2^) was 29.30 (IQR: 26.76–31.02, and the mean PSA (ng/mL) was 6.4 ng/mL (IQR: 5.15–8.6). Pathological staging revealed that 60% (n = 6) presented with organ-confined disease (T2), while 40% (n = 4) exhibited extraprostatic extension (T3).

**Table 1:** Clinical characterization of prostate cancer patients in AA men.

Characterization				Pathology		
Lab ID	HT (cm)	WT (kg)	BMI (kg/m^2^)	PSA (ng/ml)	Gleason total (Prostatectomy)	T stage
AP9	174	80.5	26.59	13	4 + 3 = 7 (GG3)	T3b
AP10	179	72.8	22.72	3.4	3 + 4 = 7 (GG1)	T2
AP11	169	82.85	29.01	9.23	3 + 4 = 7 (GG2)	T2
AP15	172	87.5	29.58	9	4 + 3 = 7 (GG3)	T2
AP18	170	105	36.33	6.4	4 + 3 = 7 (GG3)	T3b
AP19	176	94.25	30.43	4.9	3 + 4 = 7 (GG2)	T3a
AP20	178	118.55	37.42	5.9	3 + 4 = 7 (GG2)	T3
AP26	176	84.45	27.26	7.1	3 + 4 = 7 (GG2)	T2
AP30	178	75.35	23.78	4.6	3 + 4 = 7 (GG2)	T2
AP44	177	97.35	31.07	7.4	3 + 4 = 7 (GG2)	T2
Mean ± SD	174.9 ± 3.51	89.86 ± 14.15	29.42 ± 4.77	7.09 ± 2.78	NA	NA
Median [IQR]	176.0 [172.5–177.75]	85.98 [81.09–96.58]	29.30 [26.76–30.91]	6.75 [5.15–8.6]	NA	NA

Footnote: AP; African American patient, HT; Height, WT; Weight, BMI; Body Mass Index, PSA; preoperative Prostate Specific Antigen, T; Tumor. The primary tumor. GG: Grade Group) Whole-number ages 60–72. Mean ± SD: 63.3 ± 5.1; Median [IQR]: 63.0 [59.8–68.25]. IQR: Interquartile Range.

### Chromosomal hotspots and mutation density

The mutational burden was heterogeneously distributed across the cohort (Fig 1). Analysis of chromosomal distribution revealed that mutations clustered preferentially on chromosomes 1, 2, 3, 11, 17, and X (Fig 1A). When normalized for chromosome length, the highest mutation density was observed on chromosome 17, followed by chromosome 19 (Fig 1B). This density on chromosome 17 is particularly notable as it houses critical tumor suppressors, including TP53 and BRCA1, and deletions in the 17q21-22 region are established markers of aggressive disease [26]. Conversely, chromosome 19 displayed a high mutation density despite a lower total variant count, highlighting localized hotspots in regions containing KLK3 (PSA) and the pioneer factor FOXA1 [27].

**Fig 1:**
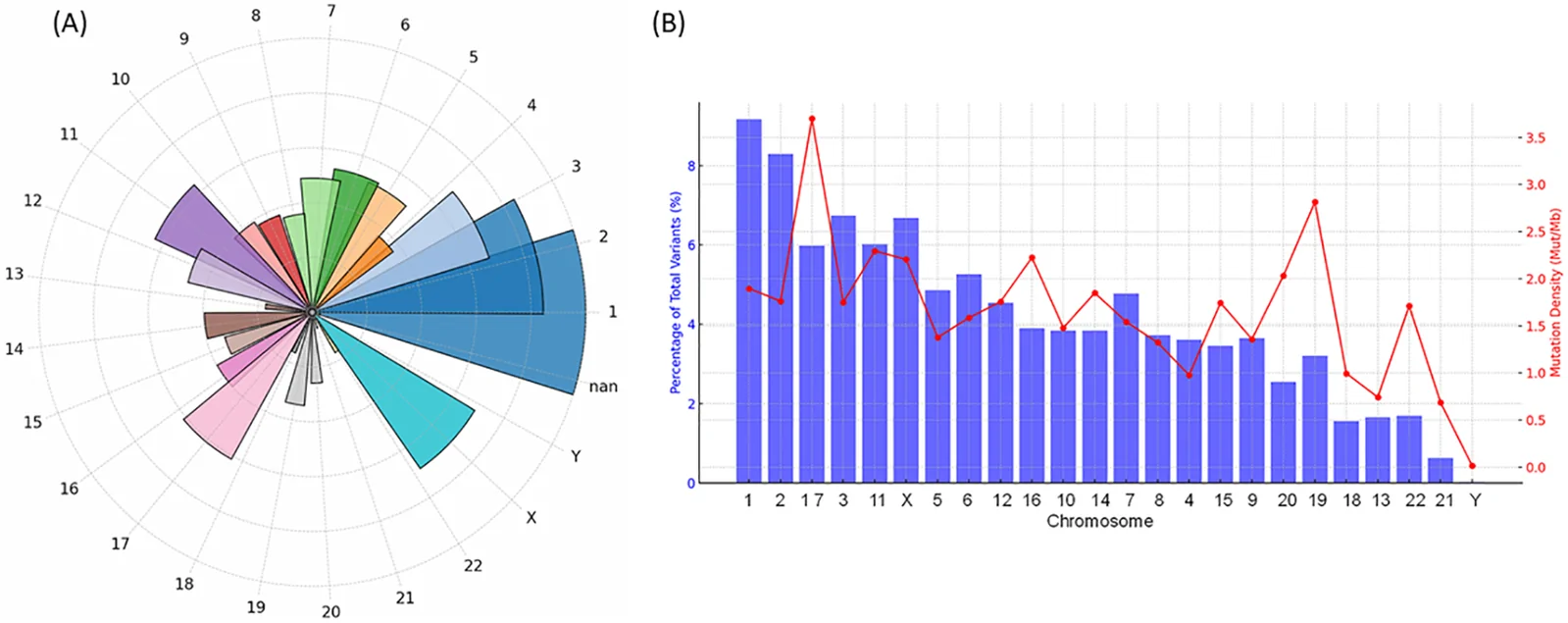
Variant counts and mutation density in chromosomes. (A) Circos plot with proportional width and height by variant count in chromosomes. The height and width of each segment represent the relative number of variants found in the individual chromosome. Chromosomes with a high number of variants have wider segments, and chromosomes with fewer variants have narrower segments. (B) Comparison of the percentage of total variants vs. mutation density. The percentage of total variants represents the proportion of mutations on a specific chromosome relative to the total number of mutations across the genome by the following formula: % variants = mutations on a chromosome/total mutations across all chromosomes X 100. Mutation density (mutations per megabase, Mut/Mb) is determined by normalizing the number of mutations by chromosome size [mutations per unit of DNA sequence (1 Mb)] by the following formula: Mutation density = mutation on a chromosome/chromosome length in megabases.

### Evidence of severe genomic instability

Beyond single-nucleotide variants, the cohort exhibited profound genomic instability. Tumor Mutational Burden (TMB) analysis identified a wide range of mutational loads, with some patients exhibiting high TMB and copy number variations (CNVs) clustered on chromosomes 1 and 2 (Fig 2A and B). TMB varied markedly across the cohort, ranging from 0.73 mutations/Mb in AP44 to 46.37 mutations/Mb in AP9.

**Fig 2:**
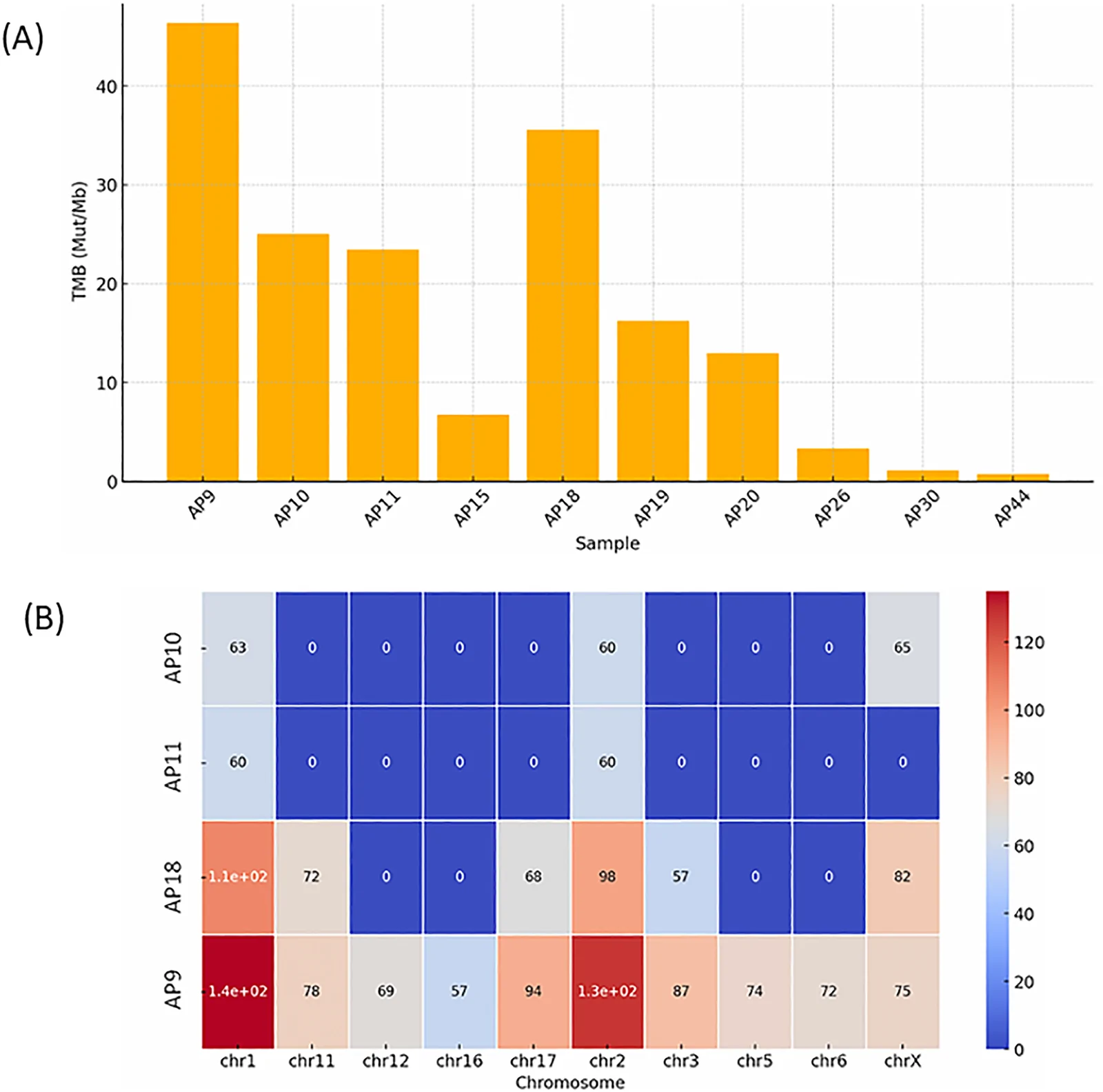
Tumor Mutational Burden (TMB) and Copy Number Variation (CNV). **(A)** TMB per sample was calculated as the number of nonsynonymous somatic mutations per megabase (Mb) of the coding genome by using the TMB Algorithm v4.0, on Reporter Software (v5.18). Differences in mutation burden were compared across patients. **(B)** Heatmap showing chromosome-level copy-number/genomic alteration counts in the four samples. Higher values indicate greater alteration burden in the indicated chromosome. CNV regions were cross-referenced with the Cosmic Cancer Gene Census using custom Python scripts. Prominent alterations were observed on chromosomes 1 and 2, particularly in AP9 and AP18. WGS reads were aligned to hs38DH/GRCh38, and CNVs were identified using CNVkit.

Further variant analysis revealed recurrent events of chromothripsis—catastrophic chromosomal shattering—particularly in high-TMB patients (Fig 3A). These events impacted genes critical for cell fate and genomic stability, including *HBS1L*, *E2F3*, and *NOTCH4*. The disruption of *E2F3*, a key regulator of cell cycle progression [28], and *NOTCH4* suggests potential therapeutic vulnerability to CDK4/6 and Notch pathway inhibitors, respectively [29]. Furthermore, we identified signatures of kataegis, or localized hypermutation, often associated with APOBEC activity and single-strand breaks (Fig 3B) [30]. These structural alterations in genes such as *TTN*, *ATRX*, and *KDM6A* are known to drive aggressive prostate cancer subtypes and underscore a phenotype of genomic chaos that may drive therapy resistance [31,32].

**Fig 3:**
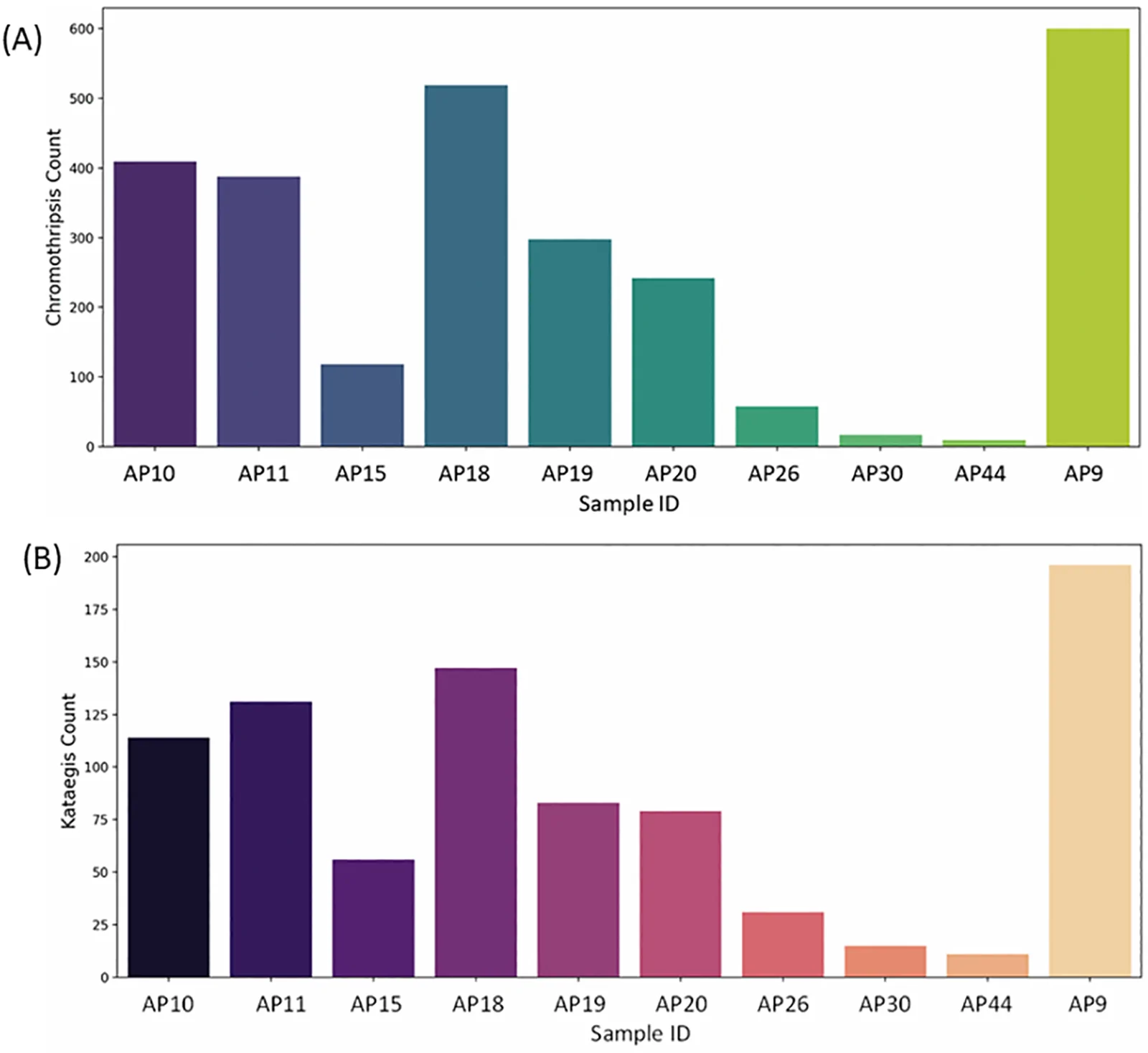
Analyses of Chromothripsis and Kataegis. **(A)** For chromothripsis, genomic rearrangements within specific chromosomal regions were detected using the following approaches: 1) Clusters of mutations within single chromosomes, 2) Large segmental deletions, duplications, and complex rearrangements, 3) Copy number variations and high mutation density. Patterns of chromothripsis and kataegis were statistically characterized using segmentation metrics and inter-mutational distances. **(B)** For the distribution of kataegis, a pattern of localized hypermutation near rearrangement breakpoints was detected using these methods: 1) Identifying regions with mutation clusters within small genomic windows, 2) Detecting localized C > T or C > G substitutions in cytosine-rich motifs, 3) Analyzing strand bias in mutation patterns.

### Recurrently mutated pathways and therapeutic targets

To identify actionable drivers, we analyzed mutation frequencies across established cancer genes (Fig 4). The landscape was dominated by alterations in chromatin remodeling and epigenetic regulation, with *KDM6A* (31.6%), *ATRX* (21.1%), *ARID1A* (7.9%), and *CHD1* (5.3%) emerging as top hits.

**Fig 4:**
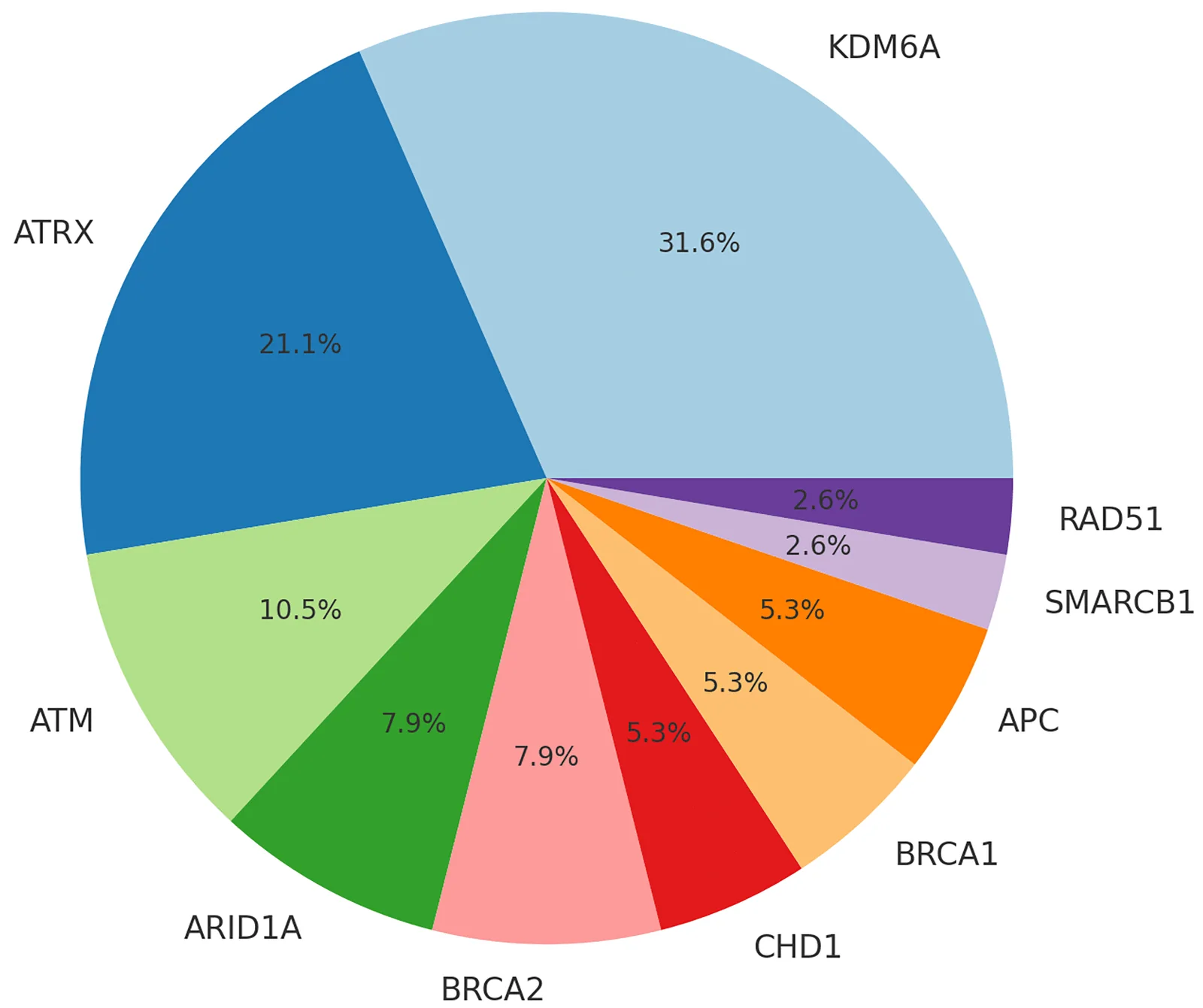
The distribution of the top 10 mutated genes. The pie chart represents the distribution of the top 10 mutated genes, illustrating their relative contribution to the total mutation burden.

Crucially, we observed significant enrichment in the DNA damage response (DDR) pathway. Mutations in *ATM* (10.5%), *BRCA2* (7.9%), *BRCA1* (5.3%), and *RAD51* (2.6%) were detected, suggesting that a subset of tumors may be sensitive to PARP inhibition. Pathway mapping confirmed that these drivers converge on critical oncogenic axes, including the PI3K/AKT/mTOR, Wnt signaling, and Androgen Receptor (AR) pathways (Fig 5A).

**Fig 5:**
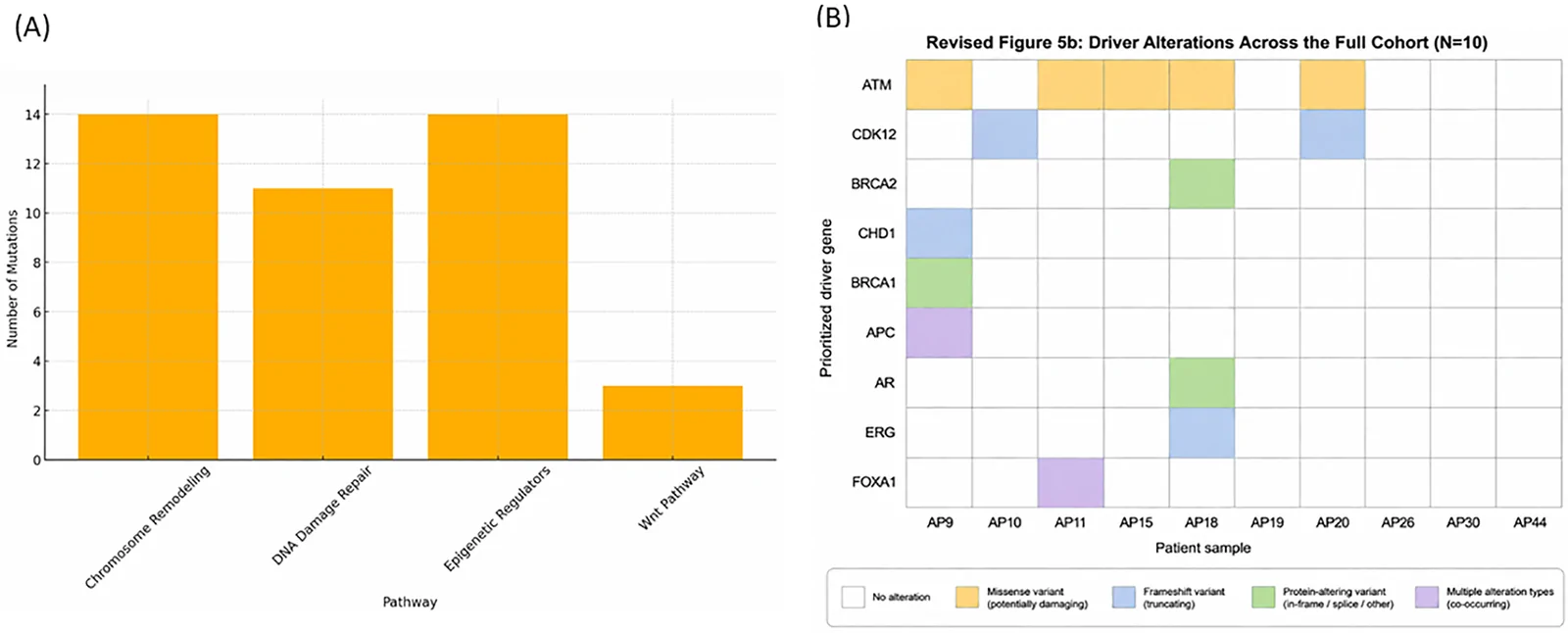
Mutation distribution across pathways and cancer driver mutations across samples. **(A)** Pathways include chromatin remodeling, DNA damage repair, epigenetic regulation, MAPK, Wnt, and PI3K across the patient datasets. **(B)** Oncoprint-style heatmap showing somatic alterations in prioritized prostate cancer driver genes across all 10 patient samples. Rows represent prioritized driver genes and columns represent individual patient samples. Colored cells denote the presence and class of a somatic alteration, whereas white cells indicate no identified alteration in the corresponding gene.

Integration of functional impact scores (SIFT/PolyPhen) prioritized likely pathogenic drivers, identifying shared variants (e.g., *ATM, CDK12*) across multiple samples, suggesting potential common drivers. Some driver genes are sample-specific, including unique candidate mutations such as *FOXA1-SSTR1* (missense variant within *FOXA1;SSTR1* structural rearrangement), which is adjacent to the breakpoint junction of the structural rearrangement involving *SSTR1* and may influence personalized therapy strategies (Fig 5B, Table 2). The prevalence of these high-impact variants in a small cohort strongly suggests that a distinct constellation of genomic alterations drives AA prostate cancer compared with European cohorts [4–11]. Alteration classes are distinguished as potentially damaging missense variants, truncating frameshift variants, other protein-altering variants (including in-frame or splice-associated alterations), and co-occurring/multiple alteration types, as indicated in the legend. The full-cohort representation highlights marked interpatient genomic heterogeneity and recurrent alterations in DNA damage-response genes, including *ATM, CDK12,* and *BRCA2*, while retaining mutation-negative samples to provide an unbiased representation of alteration frequency across the cohort.

**Table 2:** Cancer driver mutations per sample.

Gene	Chrom/Position	Top Consequence	Lab ID
*CHD1*	chr5:98894660	missense_variant	AP9
*ATM*	chr11:108343348	missense_variant	AP9
*CHD1*	chr5:98894667	missense_variant	AP9
*BRCA1*	chr17:43094396	missense_variant	AP9
*APC*	chr5:112839106	missense_variant	AP9
*CDK12*	chr17:39462342	missense_variant	AP11
*FOXA1*	chr14:37823821	missense_variant	AP11
*ATM*	chr11:108250771	missense_variant	AP11
*BRCA2*	chr13:32340382	frameshift_variant	AP18
*AR*	chrX:67643283	missense_variant	AP18
*ATM*	chr11:108343348	missense_variant	AP18
*BRCA2*	chr13:32379508	inframe_deletion	AP18
*BRCA2*	chr13:32340381	protein_altering_variant	AP18
*CDK12*	chr17:39526181	missense_variant	AP18
*CDK12*	chr17:39526183	missense_variant	AP18
*ERG*	chr21:38383860	missense_variant	AP18
*ATM*	chr11:108282861	missense_variant	AP20
*CDK12*	chr17:39524882	missense_variant	AP20

**Footnote:** *FOXA1* variant shows a structural rearrangement with the somatostatin receptor 1 (*SSTR1)* gene.

### Association of genomic instability with clinicopathologic characteristics

To explore the clinical relevance of genomic instability, we evaluated associations between genomic burden and clinicopathologic characteristics using Spearman rank correlation. Mutation-burden category was not significantly associated with preoperative PSA (ρ = 0.152, P = 0.676), Grade Group (ρ=−0.012, P = 0.974), or pathologic stage (ρ = 0.467, P = 0.174). (Fig 6).

**Fig 6:**
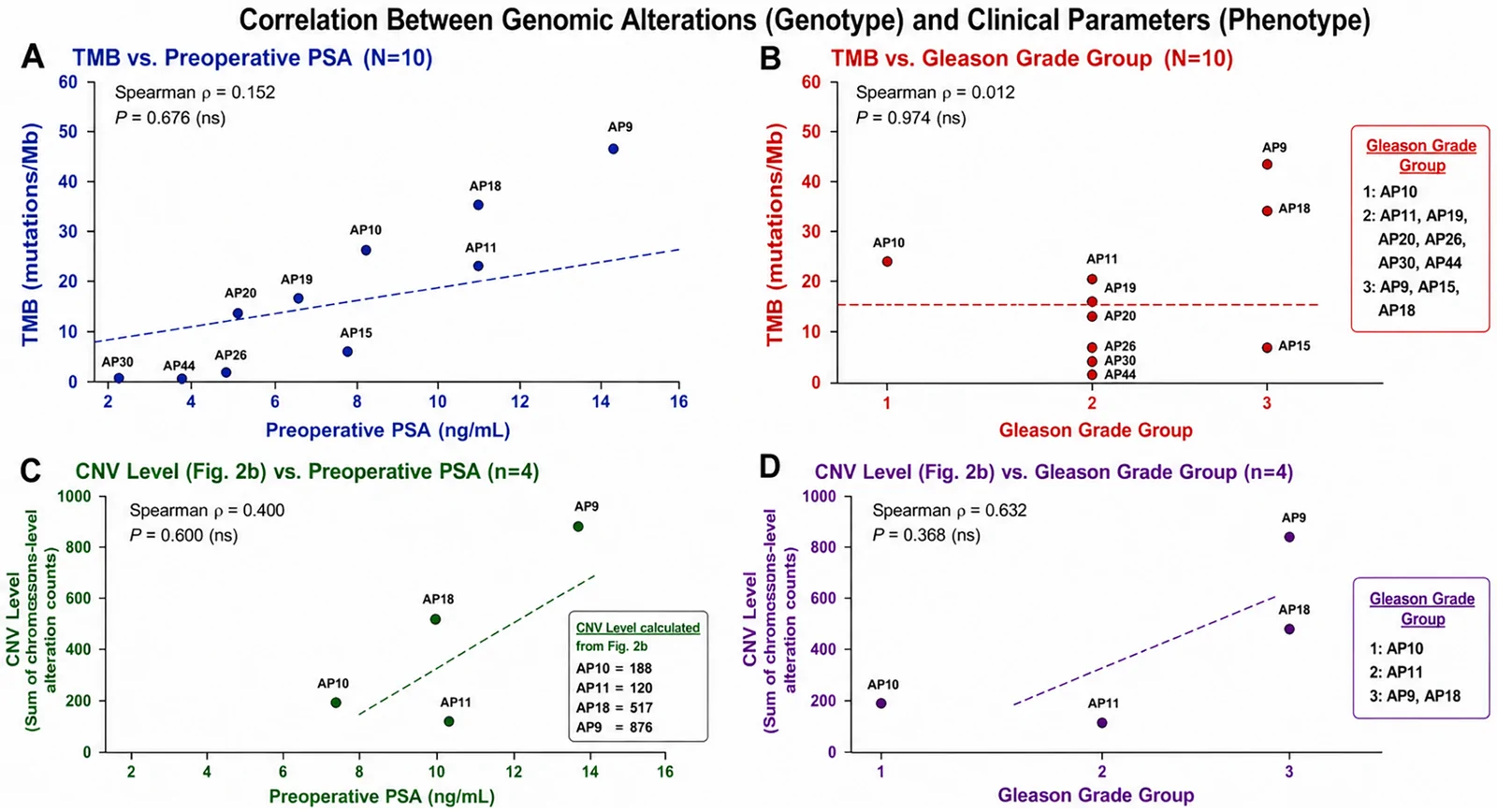
Genotype–phenotype correlation between genomic alterations and clinicopathologic characteristics. Associations between genomic alterations and clinical parameters were evaluated using two-sided Spearman rank correlation. **(A)** Relationship between tumor mutational burden (TMB; nonsynonymous somatic mutations per megabase) and preoperative PSA level across the complete cohort (N = 10). **(B)** Relationship between TMB and Gleason Grade Group across the complete cohort. **(C)** Relationship between chromosome-level copy-number variation (CNV) burden and preoperative PSA among the four patients represented in Fig 2b (AP9, AP10, AP11, and AP18). **(D)** Relationship between CNV burden and Gleason Grade Group in these four patients. Individual points represent patient samples, and dashed lines illustrate the overall direction of association. ns, not significant (P ≥ 0.05); TMB, tumor mutational burden; CNV, copy-number variation; PSA, prostate-specific antigen.

TMB demonstrated a positive association with increasing pathologic T stage (Spearman ρ = 0.012, nominal P = 0.974), while AP9 and AP18—the two T3b tumors—also exhibited the greatest chromosome-level alteration burden among samples represented in the CNV heatmap, suggesting greater somatic mutation burden in tumors with more locally advanced disease. This pattern is biologically useful: the two T3b tumors, AP9 and AP18, have among the highest TMB values, while several T2 tumors, especially AP26, AP30, and AP44, have very low burdens. TMB was not significantly correlated with preoperative PSA (ρ = 0.152, P = 0.676) or Grade Group (ρ = 0.012, P = 0.974). After Benjamini–Hochberg correction for the three TMB–clinical comparisons, this association was no longer significant at an FDR threshold of 0.05 (adjusted P = 0.113).

CNV burden in panels C and D represents the sum of chromosome-level alteration counts displayed in Fig 2b and therefore should be interpreted as an exploratory measure rather than genome-wide CNV burden. In these four patients, CNV burden showed a positive but non-significant association with PSA (ρ = 0.400, P = 0.600). A positive association with Grade Group was observed (ρ = 0.632, P = 0.368), but it did not reach statistical significance. Given the small cohort size, particularly for the CNV analysis (n = 4), these genotype–phenotype relationships are considered exploratory and hypothesis-generating. These genotype–phenotype analyses require validation in larger independent cohorts.

## Discussion

In this study, we performed whole-genome sequencing (WGS) of paired tumor and non-tumor primary cell cultures derived exclusively from African American men with prostate cancer. Our analysis revealed a complex genomic landscape characterized by significant instability—including chromothripsis and kataegis—and pathogenic mutations in critical oncogenic pathways [33,34]. A central finding of this investigation is the identification of unique chromosomal hotspots, particularly the high mutation density observed on chromosome 17, which houses critical tumor suppressors.

Clinically, the enrichment of mutations in DNA damage repair (DDR) genes (*ATM, BRCA1/2, RAD51*) and chromatin remodelers (*KDM6A, CHD1, ARID1A*) highlights specific therapeutic vulnerabilities [4]. For instance, the presence of DDR defects suggests that a subset of African American patients may benefit from PARP inhibitors (e.g., olaparib, rucaparib) [35,36]. Similarly, alterations in the *SWI/SNF* complex components *ARID1A* and *CHD1* may sensitize tumors to EZH2 inhibitors (e.g., tazemetostat), offering a targeted strategy for patients harboring these specific drivers [37,38].

We acknowledge that a cohort of 10 patients is not powered to determine population-level mutation frequencies. However, the primary objective of this study was to establish a proof of principle for a high-fidelity discovery platform tailored to precision medicine. Unlike large-scale analyses of formalin-fixed paraffin-embedded tissues, which are often compromised by cellular heterogeneity and DNA degradation, our use of pure primary cell cultures provides a high-quality genomic signal. Furthermore, the paired design—utilizing non-tumor epithelial cells from the same patient as a germline control—represents a gold standard for somatic variant calling, ensuring high confidence in the identified alterations.

Exploratory integration of genomic and clinicopathologic characteristics revealed substantial interpatient heterogeneity. Although no genotype–phenotype association reached conventional statistical significance in this small cohort, total mutational-variant burden showed a moderate positive relationship with PSA (Spearman ρ = 0.152, P = 0.676). These findings raise the possibility that genomic instability may accompany greater tumor-associated PSA burden in a subset of prostate cancers. However, because of the limited sample size and restricted clinicopathologic spectrum, these observations should be considered hypothesis-generating and require validation in larger ancestry-diverse cohorts. The lack of statistically significant associations should not be interpreted as evidence of absence of biological relationships, but rather as a reflection of the limited statistical power inherent in this discovery cohort.

Significantly, the mutational landscape observed in this cohort diverges from that reported in larger, pan-ethnic datasets, which are predominantly derived from populations of European ancestry [10,39]. The absence of certain “common” mutations and the presence of unique variants in our samples underscore the critical gap in current genomic knowledge regarding African American prostate cancer. These findings reinforce the urgent need for ancestry-specific research to prevent the widening of health disparities in the era of precision oncology.

Ultimately, these patient-derived cell models represent more than static data points; they are a renewable, functional resource. They provide a direct pathway to functionally validate novel variants and perform preclinical testing of targeted therapies, a crucial step toward developing personalized strategies for this high-risk population.

## Conclusion

This study demonstrates the feasibility and clinical utility of using WGS on matched tumor-normal primary cell cultures to identify patient-specific drivers in African American men. Despite the limited sample size, our approach enabled the high-resolution detection of functionally impactful variants in key therapeutic pathways, including DNA damage repair and chromatin remodeling. The distinct molecular landscape observed in this cohort highlights the need for ancestry-informed genomic profiling to ensure equitable access to precision medicine. These findings offer a valuable foundation for future validation studies and therapeutic matching efforts in historically underrepresented populations.

## Supporting information

S1 File Revised supporting data. (ZIP)
